# Effects of maternal obesity on the success of assisted vaginal delivery in Chinese women

**DOI:** 10.1186/s12884-018-2151-0

**Published:** 2018-12-27

**Authors:** Hongying Wu, Jiayi Yue

**Affiliations:** Department of Gynaecology and Obstetrics, Liaocheng People’s Hospital, Liaocheng, 252000 Shandong Province, People’s Republic of China

**Keywords:** Maternal obesity, Vacuum, Forceps, Assisted-vaginal delivery

## Abstract

**Background:**

We examined the influence of pre-pregnancy body weight on the rates of attempted and successfully assisted-vaginal delivery.

**Methods:**

We used 2008–2016 inpatient records including 3408 women who had singleton gestations and needed operative delivery assistance to conduct a retrospective cohort study. Patients were categorized based on pre-pregnancy BMI (normal weight = 18.5 to less than 25 or obese = 30 or greater). We used logistic regression to estimate odds ratios and 95% confidence intervals of attempted and successful forceps or vacuum-assisted vaginal delivery by body weight adjusted for marital status, age, gestational age, induction of labor, episiotomy, diabetes, and birth weight.

**Results:**

The proportion of women with attempted either vacuum or forceps was lower among women who were obese pre-pregnancy compared to women who were normal weight. Women with excessive gestational weight gain, large for gestational age neonates, and diabetes were less likely to have a vacuum-assisted or forceps-assisted vaginal delivery attempted. Conversely, women who received labor augmentation or induction, used epidural anesthesia, gained inadequate weight, and delivered a small for gestational age infant were more likely to have a vacuum-assisted or forceps-assisted vaginal delivery attempted. Compared to normal weight women, obese women who received forceps-assisted vaginal delivery were more likely to have a successful vaginal delivery.

**Conclusion:**

Women who had normal weight had higher likelihood to attempt assisted vaginal delivery compared to women who had pre-pregnancy obesity. However, when assisted vaginal delivery was attempted, success rates were higher when forceps-assisted delivery was used compared to vacuum-assisted delivery.

## Background

Delivery methods generally include vaginal delivery and cesarean section [1–3]. As an alternative delivery method, cesarean section rate usually ranges between 10 to 15%. However, in China it is as high as 50%, which is one of the highest rates in the world. Intrinsic risk is associated with cesarean sections, which not only increase the costs of postpartum care, but also cause psychological distress [4–6], and a likely lifetime risk to women in subsequent pregnancies. Hence, the Chinese National Health and Family Planning Commission has formulated a series of policies to reduce excessive cesarean section and promote vaginal delivery [7, 8].

Assisted vaginal delivery is an important measure to promote vaginal delivery of infants by using tools including forceps and vacuum extractor during the second stage of labor [9, 10]. Assisted vaginal delivery (AVD) is often considered when immediate or potential fetal compromise is expected, when there is a need to shorten the second stage of labor for maternal benefit, or when there is an inadequate progress during second stage of labor [11, 12]. It has been shown that assisted vaginal delivery can significantly lower the rate of cesarean delivery and increase both pediatric and maternal benefits, such as reducing postpartum hemorrhage, postpartum sepsis and fetal birth asphyxia [5, 10, 12, 13]. Therefore, studies on how to implement assisted vaginal delivery methods will be critical to achieve the goal of reducing excessive cesarean section and promoting vaginal delivery.

In developing Asian countries, women generally have a lower body mass index (BMI) and a smaller gestational weight gain (GWG) than those reported in developed countries in Europe and North America [14]. However, the increases in the prevalence of obesity in pregnant women has been reported in recent years in developing countries. In addition, little information has been obtained to interpret the influence of maternal pre-pregnancy GWG and/or BMI in developing Asian countries, such as China. According to the gestational weight gain recommendations issued by Institute of Medicine (IOM) in 2009, over 50% percent of childbearing-aged women who live in northern China had excessive GWG [15, 16], which is a great public health concern in China. Until now, while there are a few studies examining the influence of maternal obesity on the outcomes of delivery in Chinese women [15–18], there are no studies on the effects of maternal obesity on vaginal delivery, even though it has been shown that maternal obesity is associated with an increased rate of cesarean delivery. Further, while studies have shown that the vacuum extractor is the preferred method because it is considered as safe for the fetus and has less likelihood to cause maternal morbidity [10, 19, 20], forceps-assisted vaginal delivery is still routinely used in many areas of China, including our hospital. However, there is lack of studies on how maternal obesity affects the rates of attempted and successful vacuum extraction and/or forceps assisted vaginal delivery. Hence, our study was designed to investigate the effects of obesity status of the childbearing-aged women on attempted and successful rates of assisted vaginal delivery in China.

## Methods

### Study design

This was a retrospective study. We used data from the medical records of women who delivered from January 2008 to December 2016 in Liaocheng People’s Hospital, Liaocheng, Shandong Province in China. Data was also validated with manual chart for delivery mode, cervical dilation, and fetal station. The institutional review board approved this study.

#### Exclusion criteria

- Planned cesarean deliveries for malpresentation
- Spontaneous vaginal delivery
- Prior history of uterine surgery
- Prior cesarean delivery
- Pregnancy terminations
- Intrauterine fetal demises
- Penatal- and postnatal-diagnosed congenital anomalies
- Maternal human immunodeficiency virus.
- No documented pre-pregnancy weight, height, delivery mode, dilation, station, or potential confounders of interest including maternal age, marital status, current smoking status, race–ethnicity, primary language, parity, episiotomy, diabetes status, hypertension, receipt of prenatal care, oxytocin induction or augmentation, delivery gestational age, and neonatal birth weight (small for gestational age, appropriate for gestational age, or LGA).
- More than one pregnancy during the study period.

#### Inclusion criteria

- Singleton gestations
- 34 weeks of gestation or greater
- Eligible for a vacuum or forceps delivery
- Require operative assistance

### Abstraction of medical records

Body mass index was calculated from pre-pregnancy weight and height obtained from the medical record. Pre-pregnancy BMI was categorized as normal weight (18.5 to less than 25) or obese (30 or greater) [21]. Gestational weight gain was calculated by subtracting pre-pregnancy weight from admission weight at delivery admission or last documented prenatal visit weight. Gestational weight gain was categorized as inadequate, appropriate, or excessive considering gestational age at delivery and using the 2009 Institute of Medicine’s guidelines (Pre-pregnancy BMI-specific recommended trajectories of ranges of gain achieved by the 40th week of gestation): 25–35 pounds for women of normal weight, and 11–20 pounds for obese women [21].

We also collected Apgar score less than 7 at 1 and 5 min, and neonatal intensive care unit admission outcomes. The distribution of potential confounders by pre-pregnancy obesity status was analyzed using Chi-squared test for categorical variables and analysis of variance for continuous variables. Logistic regression models were used to estimate odds ratios (ORs) and 95% confidence intervals (CIs) for attempted and successful vacuum or forceps assisted vaginal delivery. Variables associated with delivery mode at *p* < 0.10 were included in adjusted regression models. The rates of adverse maternal and neonatal outcomes by delivery type were also compared using crude logistic regression models. All analyses were conducted using SAS 9.1.3.

## Results

**Fig. 1:**
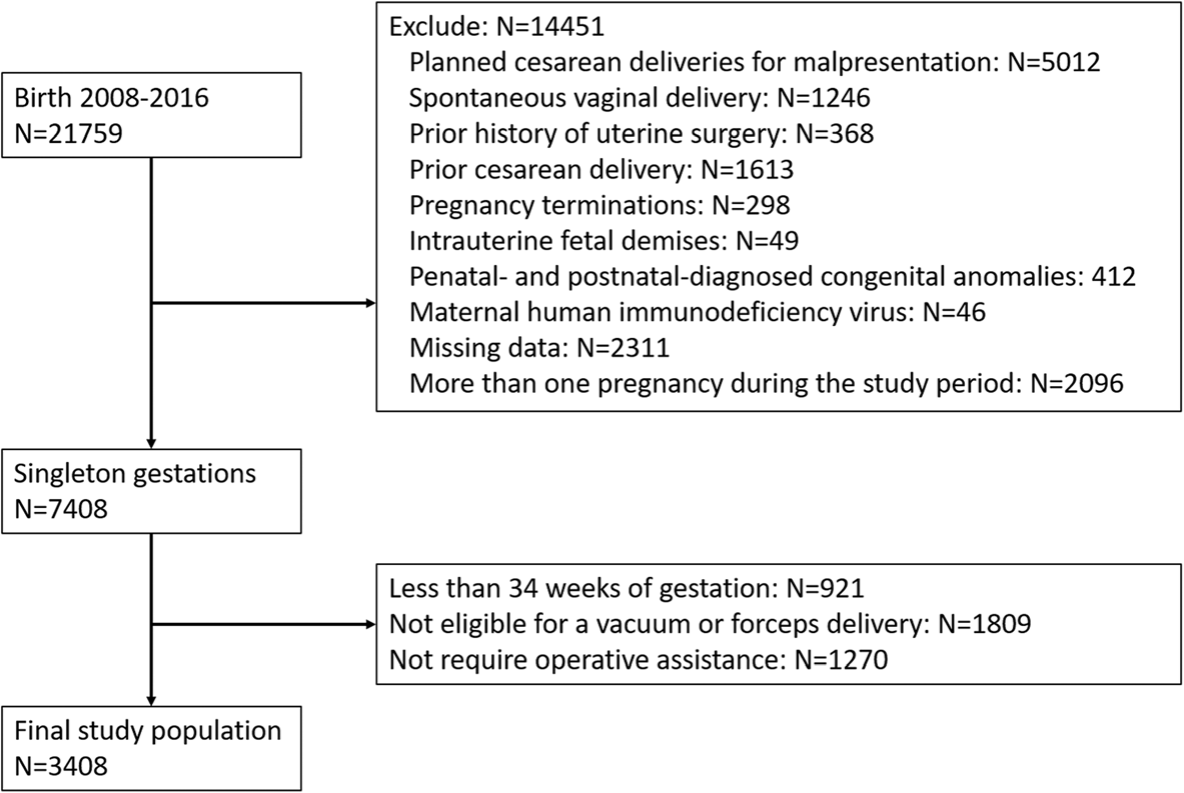
Patient Enrollment Criteria

During 2008 and 2016, there were a total of 21,759 deliveries in our hospital. We excluded 18,451 based on our exclusion and inclusion criteria. As a result, we analyzed a total of 3408 women who were normal weight, over weighted or obese pre-pregnancy and required operative delivery assistance (Fig. 1). The characteristics of the analyzed cohort are shown in Table 1. Women were predominantly married (82%) and non-smokers (89%) and nulliparous (89%). The majority of women received prenatal care (99%). Nearly half (56%) of women were normal pre-pregnancy weight, and 21% were obese. We also found that the most common comorbid conditions were hypertensive disease of pregnancy (12%) and combined pre-gestational and gestation DM (8%), with higher occurrence among obese women.

**Table 1:** Characteristics of laboring women who require operative delivery assistance, N (%) (*N* = 3408)

	Normal weight (*N* = 2059) Average BMI = 20.5	Over weight (*N* = 360) Average BMI = 26.3	Obese (*N* = 629) Average BMI = 31.2	*P*-value
Age (years)				
< 20	166 (8.1)	22 (6.1)	34 (5.5)	0.282
20–24	327 (15.9)	61 (16.9)	109 (17.3)	
25–29	580 (28.2)	109 (30.3)	187 (29.8)	
30–35	634 (30.8)	102 (28.5)	177 (28.1)	
> 35	348 (16.9)	66 (18.2)	121 (19.3)	
Smoking	187 (9.1)	12 (12.0)	72 (11.5)	0.018
Diabetes (Type I, II or GDM)	92 (4.6)	22 (6.1)	80 (12.8)	< 0.001
Hypertension: PIH, Chronic & Preeclampsia	148 (7.3)	29 (8.1)	125 (19.9)	< 0.001
Received prenatal care	2028 (98.5)	356 (99.1)	624 (99.2)	0.578
Induction	1466 (71.2)	249 (69.2)	513 (81.5)	< 0.001
Epidural Anesthesia	1812 (88.0)	308 (85.7)	560 (89.1)	0.719
Adherence to 2009 IOM guideline				
Inadequate	331 (16.1)	55 (15.2)	127 (20.2)	< 0.001
Appropriate	679 (33.0)	106 (29.5)	94 (14.9)	
Excessive	1052 (51.1)	176 (48.9)	412 (65.5)	
Gestational age				
EGA ≥34 –< 37 weeks	88 (4.3)	19 (5.2)	27 (4.3)	0.747
EGA 37+ weeks	1966 (95.5)	334 (92.9)	602 (95.7)	
Neonate weight				
LGA	222 (10.8)	39 (11.0)	41 (6.5)	0.001
AGA	1700 (82.6)	303 (84.2)	517 (82.3)	
SGA	132 (6.4)	33 (9.1)	71 (11.2)	

GDM (Gestational Diabetes Mellitus), EGA (Estimation of Gestational Age), IOM (Institute of Medicine), SGA (Small for Gestational Age), AGA (Appropriate of Gestational Age), LGA (Large for Gestational Age)

Among the women who required operative delivery assistance, 36% (*n* = 1226) had an attempted vacuum assisted vaginal delivery, 31% (*n* = 1056) had an attempted forcep-assisted vaginal delivery, and 33% (*n* = 1126) had a cesarean delivery without a trial of either assisted delivery, among which, 621 of women attempted vacuum assisted vaginal delivery and 505 of women attempted forceps assisted vaginal delivery. The proportion of women with attempted either vacuum or forceps was lower among women who were obese pre-pregnancy compared to women who were normal weight (Table 2). Women with excessive gestational weight gain, large for gestational age neonates, and diabetes were less likely to have a vacuum-assisted or forceps-assisted vaginal delivery attempted. Conversely, women who received labor augmentation or induction, used epidural anesthesia, gained inadequate weight gain, and delivered a small for gestational age infant were more likely to have a vacuum-assisted or forceps-assisted vaginal delivery attempted (Table 2).

**Table 2:** Attempted assisted vaginal delivery in relation to pre-pregnancy body weight and participant characteristics among laboring women in need of operative delivery assistance (*N* = 2282). Percentage represents the total number for each category in the final study group

Characteristics	Attempted vacuum			Attempted Forceps		
	N (%)	Crude OR [95% CI]	Adjusted OR [95% CI]	N (%)	Crude OR [95% CI]	Adjusted OR [95% CI]
Pre-pregnancy BMI (kg/m^2^)						
Normal Weight (18.5 to < 23.0)	777 (37.7)	Reference	Reference	623 (30.2)	Reference	Reference
Overweight (23.0–27.5)	145 (40.2)	0.87 [0.72–0.99]	0.88 [0.69–0.98]	131 (36.3)	0.79 [0.69–0.99]	0.76 [0.68–0.98]
Obese (≥27.5)	304 (48.3)	0.43 [0.36–0.67]*	0.42 [0.32–0.72]*	302 (48.1)	0.38 [0.31–0.59]*	0.34 [0.29–0.52]*
Age (years)						
< 20 years	88 (39.6)	1.08 [1.02–1.14]	1.15 [1.08–1.44]	78 (35.1)	0.99 [0.87–1.13]	1.08 [0.98–1.13]
20–24 years	202 (40.6)	Reference	Reference	174 (35.8)	Reference	Reference
25–29 years	354 (40.4)	0.99 [0.84–1.20]	0.97 [0.79–1.31]	305 (34.8)	0.92 [0.76–1.10]	0.93 [0.85–1.03]
30–35 years	368 (40.3)	0.88 [0.69–1.14]	0.89 [0.74–1.12]	315 (34.5)	0.89 [0.79–1.09]	0.88 [0.77–1.12]
> 35 years	214 (39.8)	0.98 [0.77–1.31]	0.98 [0.79–1.18]	184 (34.4)	0.99 [0.84–1.03]	0.98 [0.85–1.09]
Smoking Cigarettes						
No smoking	1135 (40.7)	0.98 [0.66–1.24]		969 (34.9)	0.89 [0.67–1.16]	
Smoking	91 (34.8)	Reference		87 (32.5)	Reference	
Diabetes (Type 1, 2, GDM)						
Not Diabetes	1128 (39.7)	Reference	Reference	981 (34..6)	Reference	Reference
Diabetes	98 (50.5)	0.51 [0.33–0.75]*	0.55 [0.35–0.88]*	75 (38.6)	0.68 [0.56–0.78]*	0.64 [0.54–0.76]*
Hypertension (chronic, pregnancy-induced, preeclampsia)						
Not hypertension	1150 (37.7)	Reference		987 (40.6)	Reference	
Hypertension	79 (38.3)	0.88 [0.62–1.15]		74 (37.2)	0.83 [0.61–1.16]	
Received Prenatal care						
No	17 (45.0)	1.48 [0.44–4.09]		19 (39.0)	1.48 [0.49–5.01]	
Yes	1212 (40.2)	Reference		1042 (38.2)	Reference	
Induction						
No	401 (44.7)	Reference	Reference	304 (34.2)	Reference	Reference
Yes	828 (54.9)	1.61 [1.08–2.81]*	1.77 [1.00–2.87]*	757 (45.5)	1.63 [1.08–2.83]*	1.71 [1.00–2.87]*
Epidural Anesthesia						
No	205 (15.5)	Reference	Reference	135 (10.7)	Reference	Reference
Yes	1024 (30.9)	1.26 [0.86–1.68]*	1.62 [1.12–2.39]*	926 (28.6)	1.31 [0.96–1.78]*	1.78 [1.02–2.44]*
Adherence to 2009 IOM guideline						
Inadequate	108 (88.3)	1.65 [1.10–2.41]*	1.81 [1.15–2.83]*	87 (88.1)	1.69 [1.10–2.53]*	1.88 [1.11–2.92]*
Appropriate	746 (82.1)	Reference	Reference	716 (83.1)	Reference	Reference
Excessive	345 (25.1)	0.62 [0.41–0.79]*	0.72 [0.59–1.05]*	268 (20.3)	0.61 [0.51–0.87]*	0.69 [0.59–1.01]*
Estimated Gestational age (weeks)						
EGA ≥34 – < 37	61 (51.2)	Reference		58 (47.1)	Reference	
EGA 37+	1168 (50.1)	0.89 [0.50–1.31]		1003 (48.9)	0.91 [0.60–1.51]	
Neonate weight						
LGA	182 (50.7)	3.11 [1.81–5.25]*	3.24 [1.93–6.33]*	156 (48.1)	3.28 [1.80–5.49]*	3.55 [1.91–6.03]*
AGA	982 (38.8)	Reference	Reference	859 (39.6)	Reference	Reference
SGA	65 (23.1)	0.22 [0.18–0.41]*	0.25 [0.18–0.40]*	46 (19.3)	0.29 [0.20–0.40]*	0.28 [0.18–0.45]*

Adjusted for: age category, smoking, hypertension, prenatal care, epidural, adherence to GWG, race, marital status, parity, diabetes, induction, augmentation, and estimated gestational age

^*^Significant Odds Ratios at the *p* < 0.05 level

GDM (Gestational Diabetes Mellitus), GWG (Gestational Weight Gain), IOM (Institute of Medicine), SGA (Small for Gestational Age), AGA (Appropriate of Gestational Age), LGA (Large for Gestational Age)

Among the women who had either a vacuum-assisted or forceps-assisted vaginal delivery attempt, the majority had successful assisted vaginal delivery, regardless of methods (Table 3). Compared to normal weight women, obese women who received forceps-assisted vaginal delivery were more likely to have a successful vaginal delivery. Among women who required operative assistance, there were no differences in infant 1-min and 5-min Apgar score < 7 or Neonatal Intensive Care Unit (NICU) admissions between vacuum assisted vaginal delivery, forceps assisted vaginal delivery, cesarean delivery without vacuum or forceps attempt, or failed vacuum or forceps delivery leading to cesarean (Table 4).

**Table 3:** Successful assisted vaginal delivery in relation to participant characteristics among women (*N* = 1987)

Characteristics	Successful Vacuum			Successful Forceps		
	N (%)	Crude OR [95% CI]	Adjusted OR^*^ [95% CI]	N (%)	Crude OR [95% CI]	Adjusted OR^*^ [95% CI]
Pre-pregnancy BMI (kg/m^2^)						
Normal Weight (18.5 to < 25.0)	791 (85.6)	Reference	Reference	703 (85.6)	Reference	Reference
Obese (≥30.0)	262 (86.3)	1.19 [0.59–2.28]	1.09 [0.58–2.34]	231 (96.3)	1.69 [1.07–3.18]*	1.79 [1.11–3.53]*
Age (years)						
< 20	17 (89.4)	1.11 [1.02–1.23]		11 (84.6)	0.97 [0.01–1.11]	
20–24	98 (94.2)	Reference		88 (88.8)	Reference	
25–29	312 (88.9)	0.97 [0.74–1.22]		271 (87.5)	0.85 [0.76–1.12]	
30–35	512 (84.9)	0.91 [0.69–1.21]		441 (88.6)	0.87 [0.74–1.18]	
> 35	114 (75.1)	0.99 [0.77–1.31]		123 (87.2)	0.95 [0.71–1.23]	
Smoking Cigarettes						
No Smoking	974 (85.7)	0.88 [0.68–1.06]		874 (87.9)	0.92 [0.72–1.09]	
Smoking	79 (84.0)	Reference		60 (89.5)	Reference	
Diabetes (Type 1, 2, GDM)						
Not Diabetes	994 (87.8)	Reference	Reference	889 (90.1)	Reference	Reference
Diabetes	59 (60.8)	0.49 [0.31–0.74]*	0.45 [0.27–0.68]*	45 (60.1)	0.40 [0.26–0.71]*	0.44 [0.24–0.66]*
Hypertension (chronic, pregnancy–induced, preeclampsia)						
Not hypertension	985 (85.7)	Reference		867 (87.8)	Reference	
Hypertension	68 (86.1)	0.92 [0.72–1.12]		67 (90.1)	0.85 [0.91–1.18]	
Received Prenatal Care						
No	15 (88.2)	1.08 [0.74–1.49]		17 (89.4)	1.05 [0.79–1.31]	
Yes	1038 (85.6)	Reference		917 (88.2)	Reference	
Induction						
No	345 (86.1)	Reference	Reference	265 (87.1)	Reference	Reference
Yes	708 (85.5)	1.01 [0.78–1.61]	1.07 [0.80–1.67]	668 (88.2)	1.03 [0.78–1.63]	1.01 [0.80–1.57]
Epidural						
No	185 (90.2)	Reference	Reference	115 (85.2)	Reference	Reference
Yes	868 (84.8)	1.06 [0.85–1.58]	1.07 [0.82–1.39]	819 (88.6)	1.01 [0.76–1.68]	1.08 [0.82–1.44]
Adherence To 2009 IOM Guideline						
Inadequate	94 (87.3)	1.05 [0.80–1.41]		78 (89.6)	1.09 [0.78–1.53]	
Appropriate	659 (84.9)	Reference		619 (87.7)	Reference	
Excessive	300 (86.9)	0.92 [0.71–1.12]		237 (88.4)	0.95 [0.71–1.47]	
Estimated Gestational Age						
EGA < 37	52 (85.2)	Reference		50 (86.2)	Reference	
EGA 37+	1001 (85.7)	0.99 [0.70–1.31]		884 (88.1)	0.95 [0.69–1.44]	
Neonate weight						
SGA	75 (91.7)	3.21 [1.84–5.55]*	3.14 [1.96–6.03]*	137 (87.7)	3.08 [1.89–5.79]*	3.05 [1.99–5.53]*
AGA	918 (93.0)	Reference	Reference	758 (88.2)	Reference	Reference
LGA	65 (92.3)	0.26 [0.15–0.45]*	0.24 [0.16–0.44]*	39 (84.8)	0.28 [0.17–0.44]*	0.29 [0.16–0.47]*

Adjusted for: age category, smoking, hypertension, prenatal care, epidural, adherence to GWG, race, marital status, parity, diabetes, induction, augmentation, and estimated gestational age

^*^Significant Odds Ratios at the *p* < 0.05 level

GDM (Gestational Diabetes Mellitus), GWG (Gestational Weight Gain), IOM (Institute of Medicine), SGA (Small for Gestational Age), AGA (Average for Gestational Age), LGA (Large for Gestational Age)

**Table 4:** Neonatal complications of interest by mode of delivery

	NICU Admission		Apgar Score at 1 min < 7		Apgar Score at 5 min < 7	
	N (%)	OR (95% CI)	N (%)	OR (95% CI)	N (%)	OR (95% CI)
VAVD	40 (2.7)	0.98 (0.68–1.78)	217 (20.1)	1.05 (0.75–1.38)	41 (2.9)	1.10 (0.56–2.85)
FAVD	34 (3.2)	1.03 (0.71–1.86)	221 (21.3)	1.01 (0.68–1.15)	39 (2.4)	1.15 (0.66–2.95)
CD	11 (2.9)	Ref.	88 (19.7)	Ref.	11 (2.6)	Ref.
CD after failed VAVD	3 (3.8)	1.42 (0.39–5.15)	21 (28.8)	1.33 (0.74–2.93)	4 (5.2)	1.43 (0.65–6.87)
CD after failed FAVD	2 (3.1)	1.32 (0.39–5.15)	24 (29.6)	1.43 (0.84–2.83)	5 (5.8)	1.40 (0.61–6.79)

CD (Cesarean Delivery), VAVD (vacuum-assisted vaginal delivery), FVAD (forceps-assisted vaginal delivery), NICU (Neonatal Intensive Care Unit)

## Discussion

We found that pre-pregnancy obesity reduced the likelihood in Chinese women to attempt vacuum or forceps-assisted vaginal delivery. Further, we observed higher success rates only when forceps-assisted delivery was attempted. In addition, we found that among women requiring operative delivery assistance, Apgar scores or NICU admissions were not affected by vacuum or forceps-assisted vaginal delivery, relative to cesarean delivery without assisted-vaginal delivery attempt or cesarean delivery after failed assisted vaginal delivery. These results suggested that attempting vacuum or forceps assisted vaginal delivery might not be a risky factor in certain delivery outcomes. However, it has been shown that obese women have increased risks of large for gestational age neonates and associated shoulder dystocia [18]. Also, perineal lacerations and shoulder dystocia are known risks of vacuum-assisted vaginal delivery [16, 17]. Studies have shown that women who underwent a vacuum assisted vaginal delivery had the risk rates of 3rd or 4th degree laceration (12.3%) and shoulder dystocia (2.5%) and risk rates increased relative to those who had cesarean delivery without attempted vaginally delivery [22]. Hence, clinician may always need to consider these complications deterring vacuum-or forceps assisted vaginal delivery attempts. However, this concern would need to be balanced against the significant morbidity accompanying cesarean delivery in women with obesity.

Our finding of lower odds of attempted assisted vaginal delivery in obese women is consistent with a recent study but is contradicted with previous studies [23, 24]. One of the possible reasons is the different rates of cesarean delivery. A recent prospective cohort study of Norwegian women found 50% higher rates of vacuum-assisted vaginal delivery among women with class III obesity compared to normal weight women [25]. However, a recent study in US showed lower odds of attempted assisted vaginal delivery in obese women, similar to our findings [26]. It should be noted that rates of cesarean delivery in Norway were much lower than those in the US and China during the study period [27]. Therefore, it is possible that physicians may prefer to use assisted vaginal deliveries in women with obesity, when the rate of cesarean deliveries is low. Also, obesity is associated with several health conditions, including hypertensive disease of pregnancy, chronic hypertensive disease, and gestational diabetes, which are known risk factors that are associated with higher cesarean rates. In our study, 21% of women were obese, which is similar to that in US study (18%), but much higher than that in Norway study (8%).

Another interesting finding of our study is that obese women have higher chances of success with forceps compared to normal weight women. Previous study showed that the use of forceps was associated with a higher success rate than the vacuum, for both occiput anterior and posterior positions [28]. It has previously been found that obese and short women are at greater risk of having more difficult labors [29, 30] and of occiput posterior delivery [31–33]. Hence, it is likely that forceps assisted vaginal delivery is particularly effective for obese women with occiput posterior position.

Our study has several limitations. We did not collect adequate information from medical comorbidities. Particularly, some comorbidities are associated with recommendations to shorten second stage labor, such as valvular disease and chronic obstructive pulmonary disease [16]. We might misclassify the pre-pregnancy weight status, because it was either self-reported or was measured during early prenatal care. However, it should be noted that studies shave reported that self-reported pre-pregnancy weight is highly correlated with clinically measured pre-pregnancy weight (*r* = 0.99) [34, 35], indicating that the rate of misclassification of the pre-pregnancy weight status would be low, if there is any in our study, and unlikely to alter our findings. Further our studies did not classify obesity status. A larger sample of obese women in our follow-up study will evaluate the relationship of classes of obesity and rates to attempt and of successful assisted-vaginal delivery. Finally, both vacuum and forceps extractors are safe instruments and have been used routinely for assisted-vaginal delivery. In clinical practice, while women were selected on an individualized basis and decision was made based on individual physician, the skills of operators would certainly also have an influence on the decision of women to attempt an assisted-vaginal delivery and the choice of vacuum or forceps instrument. The forceps-assisted vaginal delivery has been adopted in our hospital for a much longer time than vacuum-assisted vaginal delivery. Hence, it should be noted that the experience and skills of operators may also help increase the success rate of forceps-assisted vaginal delivery.

## Conclusion

In conclusion, our study reported that among Chinese women in need of operative delivery assistance, pre-pregnancy obesity may reduce the likelihood of an attempt to have assisted vaginal delivery. However, if assisted vaginal delivery was attempted, forceps-assisted vaginal delivery was associated with higher rate of success. This is consistent with previous reports that forceps are more likely to be used in primigravidas and less likely to fail [13, 28]. However, the risks accompanying forceps and even vacuum extraction should also be considered. Particularly, forceps-assisted vaginal delivery is reported to be associated with increased maternal morbidity and enhanced risks for facial nerve palsies and minor facial abrasions [13]. When in the clinical practice, the application of vacuum or forceps-assisted vaginal delivery in obese population should also be balanced with the higher surgical risk and morbidity of cesarean delivery [18, 24]. Hence, a more comprehensive guideline will be critical for clinicians to choose operative assistance for clinically appropriate laboring women with obesity. Future perspective studies will be necessary to examine the effects of such guidelines on the safety and efficacy in promoting vaginal delivery in a larger population.

## Abbreviations

cis: Confidence intervals
ors: Odds ratios
